# Establishment of an Animal Model Scheme of *Strongyloides stercoralis*-Infected *Meriones meridianus*

**DOI:** 10.3390/pathogens12111285

**Published:** 2023-10-26

**Authors:** Huan Zhou, Jinyang Hu, Taoxun Zhou, Ying Zhang, Peixi Qin, Biying Zhang, Rui Wang, Xiaoping Luo, Min Hu

**Affiliations:** 1 National Key Laboratory of Agricultural Microbiology, College of Veterinary Medicine, Huazhong Agricultural University, Wuhan 430070, China; hujinyang@webmail.hzau.edu.cn (J.H.); taoxunz@webmail.hzau.edu.cn (T.Z.); 15271946560@139.com (Y.Z.); peixiqin@webmail.hzau.edu.cn (P.Q.); zhangbingbing233@163.com (B.Z.); 2 School of Life Sciences, Henan University, Kaifeng 475004, China; 3 College of Veterinary Medicine, Inner Mongolia Agricultural University, Hohhot 010030, China; 4 Inner Mongolia Academy of Agriculture and Animal Husbandry Sciences, Hohhot 010030, China

**Keywords:** *Strongyloides stercoralis*, *Meriones meridianus*, animal model

## Abstract

Studying parasitic nematodes, which generate a massive hazard to animal health, is more difficult than studying free-living nematodes as appropriate animal models are essential, and the relationship between parasites and hosts is extremely complex. *Strongyloides stercoralis* is an intestinal nematode parasite that mainly infects dogs, humans and other primates. Currently, *S. stercoralis* worms needed for research mainly rely on their natural host, the dog. This study explored a method of using *Meriones meridianus* as a model for *S. stercoralis.* The immunosuppressed *M. meridianus* were infected with *S*. *stercoralis* subcutaneously, and post-parasitic, first-stage larvae (PP L1) were detected in the faeces, with more larvae in female gerbils. In addition, parasitic females (PFs), third-stage larvae (L3s) and rhabditiform larvae were found primarily in the small intestines and lungs of infected gerbils. The PFs and auto-infective third-stage larvae (aL3s) obtained from *M. meridianus* are morphologically identical to those obtained from beagles and *Meriones unguiculatus*. Moreover, the infection of *S. stercoralis* caused changes to biochemical indicators in the serum and in the physiology of *M. meridianus*. The results demonstrated that *M. meridianus* can be infected by *S. stercoralis*, and this model provides a great tool for exploring the biological processes of this parasite and its interaction with the host.

## 1. Introduction

Infections caused by parasitic helminths are among the most prevalent infections in animals and humans [1,2]. Strongyloidiasis, caused by nematodes of *Strongyloides spp.*, is one of the most important zoonotic parasitic infections shared by humans and dogs. *Strongyloides stercoralis* is a common, widely distributed and highly harmful intestinal nematode parasite that primarily infects dogs, humans and some primates [3,4,5,6]. It is mainly distributed in tropical and subtropical regions [6,7,8] and causes strongyloidiasis, which is a soil-transmitted helminthiasis (STH) that sometimes is described as one of the most neglected tropical diseases (NTDs) [9]. An acute infection, which is more harmful to the host and less common, is mainly due to exposure to a large number of infective third-stage larvae (iL3s) at one time [6]. After skin penetration, the migration of many larvae into the lungs, liver and intestines of a patient causes prominent symptoms. In severe cases, the worms multiply rapidly and affect multiple organs, which eventually leads to death, especially when the immunity of a patient is compromised [10,11]. Under some conditions associated with being immunocompromised, the auto-infective cycle of *S. stercoralis* can become amplified to hyperinfection syndrome, a potentially fatal disease characterized by increased numbers of infective filariform larvae in the stool and sputum, thereby, facilitating parasite transmission [10]. The increasing risks of zoonotic strongyloidiasis suggest the need for the rigorous control of the parasite in humans and dogs, as well as intensive biological research into the pathogen, *S. stercoralis.*

The similar morphologies of a free-living female (FL Female) of *S. stercoralis* and a hermaphrodite of the free-living nematode *Caenorhabditis elegans* make *S. stercoralis* a powerful model organism for studying parasitic nematode biology in that the extensive knowledge on the molecular biology of *C. elegans* and its useful genetic manipulation techniques can be applied to *S. stercoralis* [12]. The difficulty of studying *S. stercoralis* is that all of the progenies of FL Females are fated to become iL3s, which must infect their host to develop further. Consequently, *S. stercoralis* is capable of undergoing one generation of development outside of the host, and host animals are required for maintaining the worms continuously in the laboratory [12]. In recent years, researchers have studied the function of key genes in *S. stercoralis* through a variety of methods, but they can only explore the influence of genes on the developmental stages of larvae in vitro [12,13,14,15], not on the developmental stages of *S. stercoralis* in the host. In addition, transgenic larvae obtained by transgenic technology in vitro cannot be expanded by the host, and it is difficult to obtain a large number of larvae for RNA sequencing. Thus, research on *S. stercoralis* has not been able to make breakthroughs.

When studying strongyloidiasis, beagles and patas monkeys are commonly used as the traditional models [12,13,14,15]. However, these hosts are large in size, difficult to handle, expensive to maintain and a source of ethical concern, which limit their use as experimental animals in biological studies. To overcome this hurdle, efforts have been made in establishing *S. stercoralis* infections in many small laboratory animals, including mice, rats, guinea pigs and rabbits, but no success has been obtained as they were not susceptible to *S. stercoralis* [16]. Nevertheless, an experimental animal model of *S. stercoralis* was successfully established in *Meriones unguiculatus* in the 1990s [17,18,19]. More recently, a highly immunocompromised strain of mice, NOD.Cg-Prkdc^scid^Il2rg^tm1Wjl^/SzJ (NSG), has been successfully established as a small animal model for *S. stercoralis* infections [20,21]. However, this strain of mice has some notable defects, such as harsh feeding conditions, and the number of worms recovered from the intestines of infected mice treated without methylprednisolone acetate (MPA) was low [20]. In summary, scientists still lack a suitable economical and practical small animal models for *S. stercoralis*.

*Strongyloides stercoralis* from both dogs and humans can produce stable infections in *M. unguiculatus,* which is a vulnerable animal and not suitable as an animal model [18,22], but the susceptibility of *M. unguiculatus* to *S. stercoralis* indicated that gerbils have a strong capability for establishing an *S. stercoralis* infection. Another species of gerbil, *Meriones meridianus,* is widely distributed in Eastern Iran, Northwest China, Russia, Mongolia and the Northern Caucasus [23,24,25]. Studying *M. meridianus* as an animal model in their native area has many advantages, including convenient feeding, easy domestication, rapid breeding and a strong disease resistance [26,27,28]. In addition, *M. meridianus* does not show density-dependent and spousal-fixing reproduction, and their reproduction rates, both in the laboratory and in the wild, are high in their native area, regardless of population size and density [26,29,30]. In addition, *M. meridianus* is a proper research model for animal behaviour, physiological ecology and any other fields based on the above-mentioned advantages [25,26,31,32]. Furthermore, *M. meridianus* has been used as an animal model in several parasitological studies: *Leishmaniae* [31], *Echinococcus multilocularis* [32,33] and *Echinococcus granulosus* [32]. However, so far, the research on establishing an *S. stercoralis* infection in *M. meridianus* is missing. Here, the ability of *S. stercoralis* to establish an infection in *M. meridianus* was assessed and confirmed, which reinforce the potential of *M. meridianus* as an animal model of parasitic nematodes.

## 2. Materials and Methods

### 2.1. Experimental Animals

All animal experiments were conducted in compliance with guidelines proposed by the Administration of Affairs Concerning Experimental Animals of P.R. China and according to the protocol (Permit No. SYXK-2015-0029) approved by the Animal Ethics and Animal Experimentation Committee of Hubei Province. The *S. stercoralis* UPD strain (University of Pennsylvania, Dog), a subclinical infection in laboratory dogs, was maintained in beagles as previously described [12]. The *M. meridianus* were a gift from Inner Mongolia Agricultural University and were kept as 2–5 animals per cage at 25 °C. The gerbils were 6–10 weeks old and weighed between 30–60 g at the start of the experiments. The dogs and gerbils were fed with standard laboratory chow and kept at a constant temperature of 26 °C.

### 2.2. Parasite Maintenance and Culture

iL3s were isolated from the faeces, which have been cultured for more than 7 days at 22 °C, of prednisolone-treated beagles infected with *S. stercoralis* and collected using a Baermann apparatus. The mixture, which was made by mixing faeces freshly collected from the infected dog with charcoal [12], was held at 22 °C for more than 10 days. The worms were washed with BU buffer (50 mM Na_2_HPO_4_, 22 mM KH_2_PO_4_, 70 mM NaCl) 5 times. Then, iL3s were counted and mixed with gentamicin sulphate for an immediate subsequent infection.

### 2.3. Gerbil and Dog Infection

The *M. meridianus*, including 4 females and 6 males, were divided into four groups. Group 1 (three male gerbils) was infected, but not immunosuppressed, to test whether *M. meridianus* can be successfully infected by *S. stercoralis* without immunosuppression. The other three groups were the infected and immunosuppressed. Group 2 (one female gerbil) aimed to test whether *S. stercoralis* can perform a complete life cycle in *M. meridianus*. Group 3 (three female gerbils) and group 4 (three male gerbils) were designed to assess whether gender affects the infection efficiency of *S. stercoralis.* For immunosuppression, *M. meridianus* (Group 2, Group 3 and Group 4) and *M. unguiculatus* (n = 4) were given an intramuscular injection of 100 µL of methylprednisolone acetate (MPA, 2.5 mg per gerbil each time) two days before the infection and twice a week after the infection, but *M. meridianus* of Group 1 were not. Immunosuppressed *M. meridianus* and *M. unguiculatus* were infected by subcutaneously injecting a suspension of 1000 iL3s in 200 ul of gentamicin sulphate into the nape. Each gerbil was placed into a 1 animal/cage after the infection at room temperature (25 °C). The *Strongyloides stercoralis* infection of the beagles was carried out according to the standard method [12].

### 2.4. Stool Examination and Necropsy Procedures

To observe the incubation period and evaluate the infection efficiency, a stool examination of the number of post-parasitic first-stage larvae (PP L1s) from infected gerbils’ faeces was performed. A piece of cardboard soaked in water was placed under the cage to keep the faeces that fall onto the cardboard moist. After 12 h, the collected and weighed faeces from the cardboard were wrapped in a double layer of gauze and placed in a Baermann apparatus. Tap water was gently poured at 42 °C, and the device was kept at room temperature for 4 h. The contents from the Baermann funnels were collected every hour to avoid hypoxic stress to the isolated PP L1s.

The *M. meridianus* in Group 2 was euthanised via a cervical dislocation after hunger breeding for 24 h on day 21 after the infection, and the digestive tract (stomach, oesophagus and small and large intestines) was slit longitudinally. The contents were washed away cautiously. The slit digestive tract of *M. meridianus* (Group 2), which was tied with a rope, was submerged in 50 mL cylinders of DMEM (Dulbecco’s Modified Eagle Medium) for 3.5 h at 37 °C. At the same time, the shaved skin, bones, muscles and thoracic and abdominal organs were wrapped with gauze and soaked in BU buffer for 4 h. To examine the adult worms and larvae from the intestines, lungs and stomach, a small amount of liquid that passed through the funnel was set onto a flat dish and placed under the microscope (SZX16 Olympus) for observations. The same method was used to dissect *M. unguiculatus* and obtain the worms. The beagles, which developed an acute infection with *S. stercoralis,* were euthanized for moral reasons using IV administration of an overdose of pentobarbital sodium. The intestines were slit longitudinally and cleansed cautiously, and the worms were obtained from the intestines using a Baermann apparatus.

### 2.5. Histology

The duodenum of *M. meridianus* was fixed in 2% paraformaldehyde for 16 h at 4 °C, processed and embedded into paraffin blocks, according to routine procedures. After that, contiguous sections were taken for haematoxylin-eosin staining (HE) preparations. Sections were stained with haematoxylin and eosin using standard pathologic procedures. Briefly, sections were deparaffinized in xylene three times for 3 min each and rehydrated with successive washes (1 min) in 100%, 100%, 80%, and 70% ethanol. They were then stained with haematoxylin for 5 min and rinsed with distilled water, 0.1% hydrochloric acid in 50% ethanol and tap water for 2 min each. Next, the sections were rinsed with 0.5% ammonia for 1 min, stained with eosin for 1 min and rinsed again with distilled water. The slides were then successively dehydrated with 95% and 100% ethanol, followed by xylene two times for 5 min each and mounted with coverslips.

### 2.6. Morphology and Statistics

The specimens, which had been washed with BU buffer, were transferred to Nematode Growth Medium (NGM) agar plates. The immobilization was performed and anaesthesia was administered using the methods as previously described [14]. In brief, parasitic females (PFs) and auto-infective third-stage larvae (aL3s) were transferred to a 2% agarose pad (Lonza, Switzerland) containing 5 μL–10 μL of a levamisole solution (100 mM, Sigma-Aldrich, USA) for the immobilization. Then, a coverslip stained with petroleum jelly on all four sides was placed gently over the larvae, and the locations of the larvae under the microscope were marked. Morphological observations on the worms were made with the Olympus BX53 compound microscope equipped with Nomarski Differential Interference Contrast (DIC) optics. The body lengths and widths of PFs and post-parasitic third-stage larvae (PP L3) were measured using the ImageJ software: http://rsb.info.nih.gov/ij/ (accessed on 12 December 2022), and more than 30 PF and PP L3 were measured, respectively. The values for all the data were analysed using a one-way ANOVA with Bonferroni’s multiple comparisons test for the multiple groups and drawn in Prism (GraphPad Software, Inc., La Jolla, CA, USA). *p* ≤ 0.05 was the criterion for significance.

### 2.7. Haematological Assessments

Blood was collected from the eyeball of gerbils (49 days after infection for Group 1 and Group 4) anesthetized with ether for about 30 s, and the anticoagulant, EDTA-K2, was immediately added. The serum was obtained by centrifuging the blood, which had not been treated with the anticoagulant, at 3000 rpm for 10 min. Twenty-three blood parameters, including the white blood cell (WBC) count, lymphocyte ratio (Lym#), neutrophil ratio (Neu#), eosinophil ratio (Eos#), basophil ratio (Bas#), haemoglobin (HGB), red blood cell (RBC) count, haematocrit (HCT), mean corpuscular volume (MCV), mean corpuscular haemoglobin (MCH), mean corpuscular haemoglobin concentration (MCHC), blood platelet (PLT) and plateletcrit (PCT), were detected using a fully automated blood cell analyser BC-5500. The total protein (TP), albumin (ALB), total bilirubin (T-Bil), triglyceride (TG), alanine aminotransferase (ALT), urea (UA) and triglyceride (TG) were detected by the Hitachi 7020 automatic biochemical analyser.

## 3. Results

### 3.1. Both Female and Male M. meridianus Can Be Infected by S. stercoralis

**Figure 1:**
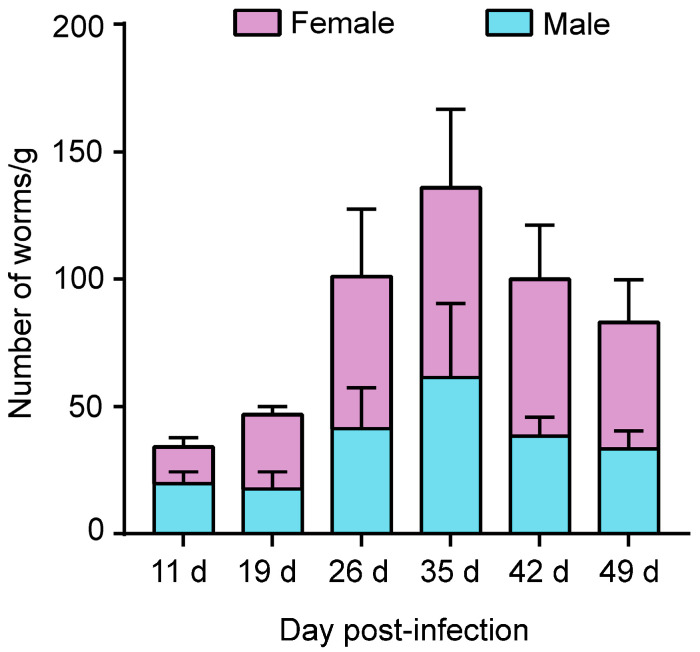
Number of post-parasitic first-stage larvae (PP L1s) of *Strongyloides stercoralis* recovered from faeces of female and male *Meriones meridianus*. There was no difference in the number of worms between the faeces of male and female *M. meridianus*. The abscissa is the time of sampling, and the ordinate is the number of larvae per gram of faeces. Three gerbils were used in each group.

Six immunosuppressed *M. meridianus* from the infected groups (Groups 3 and 4) were subcutaneously injected with 1000 iL3s individually. There were no larvae in the faeces within 10 days of the infection, and on day 11, after the infection, larvae were detected in the faeces of both female and male gerbils; however, the numbers were only 18 and 20 per gram of faeces, respectively. The number of larvae in the faeces was then examined on days 19, 26, 35, 42, and 49 after the infection. The amounts of PP L1 found in the faeces of both male and female gerbils increased rapidly over time, within 35 days of the infection, and after that, they dropped slightly (Figure 1). On average, more PP L1s were detected in the infected female *M. meridianus* than in the male gerbils, even though there was no significant difference noted (Figure 1). No PP L1s were found in the faeces of the three non-immunosuppressed *M. meridianus* (Group 1) injected with 1000 iL3s of *S. stercoralis*.

### 3.2. Strongyloides stercoralis Complete Their Life Cycle in M. meridianus

**Figure 2:**
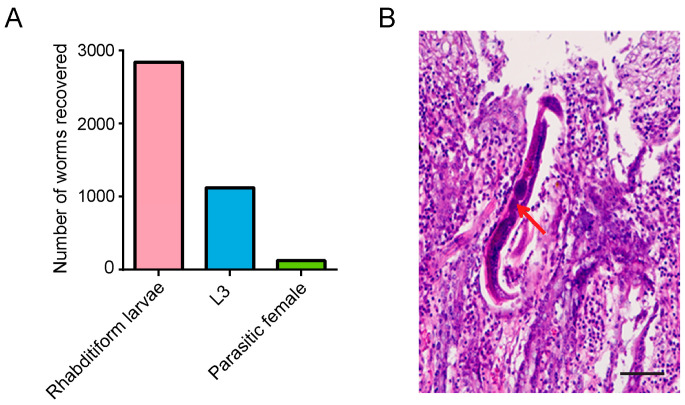
*Strongyloides stercoralis* within the tissues of *Meriones meridianus***.** (**A**) Parasitic female (PF), third-stage larvae (L3s) and rhabditiform larvae (L1 + L2) were isolated from *Meriones meridianus* infected with *S. stercoralis*. *Meriones meridianus* infected with 1000 iL3s were dissected 21 days after the infection, and 126 PF, 1120 L3 and 2842 rhabditiform larvae were isolated from the gerbil. (**B**) Distribution of *S. stercoralis* in the intestine of *M. meridianus*. HE staining shows that adult worms were observed and several eggs were present in the adult gonad. Red arrow: eggs inside the parasitic female (PF), Bar = 100 μm.

The *M. meridianus* in Group 2 was killed and dissected on day 21 after the infection. A total of 126 PFs were recovered from the small intestine, and some worms were also found in other tissues. In addition, nearly 1000 of L3s were collected from all tissues (Figure 2A). The discovery of PFs in the intestine and L3s in the tissues indicates that subcutaneously injected iL3s successfully migrated to the intestine and developed into adult worms in *M. meridianus*. Several unfertilized eggs were observed in the gonads of PFs, and these eggs could successfully hatch and develop into first-stage larvae (L1) and second-stage larvae (L2) in the small intestine of *M. meridianus*. Nearly 3000 rhabditiform larvae (L1s and L2s) were collected from the gerbil (Figure 2A). To determine the distribution of *S. stercoralis* within the small intestinal tissue, we performed extensive sections of the small intestine as well as HE staining. Adult worms were observed and several eggs were present in the adult gonad (Figure 2B).

### 3.3. Morphometric Features of S. stercoralis from M. meridianus

The body length and maximum diameter of the PFs and PP L3s from *M. meridianus* are presented in Table 1. The average length of the PF is 2.14 mm ± 0.263 mm and that of the PP L3 is approximately 0.544 mm ± 0.181 mm, which is similar to the PFs and PP L3s collected from dogs and *M. unguiculatus*, respectively (Table 1). There was no difference in the body length and maximum diameter between the PFs or PP L3s from *M. meridianus*, *M. unguiculatus* and beagles.

**Figure 3:**
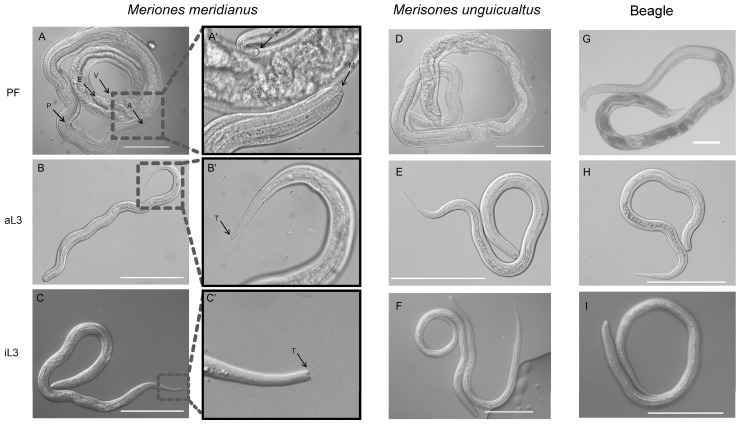
Morphological characteristics of a parasitic female (PF) and auto-infective third-stage larva (aL3) of *Strongyloides stercoralis* collected from an infected *Meriones meridianus*, *Meriones unguiculatus* and beagle. Differential Interference Contrast (DIC) of a PF and an aL3 of *S. stercoralis*. (**A**,**A’**) A PF worm from *M. meridianus* and the magnified display of its anterior end and tail. (**B**,**B’**) An aL3 larva from *M. meridianus* and the magnified display of its tail. (**C**,**C’**) An iL3 larva from *M. meridianus* and the magnified display of its tail. (**D**–**F**) PF, aL3 and iL3 from *M. unguiculatus*. (**G**–**I**) PF, aL3 and iL3 from a beagle. P: pharynx; E: egg; A: anterior end; T: tail; M: mouth; V: vulva. Bar = 100 μm.

Our observation of the morphology of the PF and aL3 from *M. meridianus* using the method described previously [14] found that the anterior end (A) of the PF is transparent and the length of the pharynx is close to one-half of the body length (Figure 3A,A’). The vaginal opening of the double uterus, the vulva (V), is located two-thirds from the anterior end and looks like a “crater”. The stratum corneum is smooth and covers the whole body; the process of exfoliation is observed occasionally. The gonad is wrinkled, and the eggs, which are slightly longer than those from the FL females, are elliptical. In addition, there are some differences in morphology between an iL3 and aL3. An aL3 could be distinguished from an iL3 based on the size and shape of its tail. Compared with an iL3, an aL3 is thicker and shorter, and the tail of an aL3 is bluntly pointed, which is different from the bifid tail of iL3 (Figure 3B–C’). The PF, aL3 and iL3 worms from *M. meridianus* have the same morphology as those from *M. unguiculatus* and a beagle (Figure 3A–I).

### 3.4. Changes to Physiological and Biochemical Parameters of M. meridianus Infected with S. stercoralis

The changes in biochemical parameters in the serum of *M. meridianus* caused by the infection mainly included a significant increase of alanine aminotransferase (ALT), which is mainly present in liver tissue. Its elevation indicates abnormal liver function, such as acute hepatitis. ALT increased from 46 U/L to 201 U/L after infection with *S. stercoralis* (a 4.37-fold increase). In addition, another parameter associated with acute infection, triglyceride (TG), was also significantly elevated to 16.69 mmol/L, which was 19.87 times higher than that of unsuccessfully infected gerbils. The physiological index showed that compared with Group 1, the number of red blood cells (RBC) in the successfully infected gerbil was reduced from 6.13 × 10^12^/L to 4.68 × 10^12^/L, but the Haematocrit (HCT) had no significant change. Furthermore, another physiological index, blood platelet (PLT), which is associated with acute hepatitis, increased to 5.8 times more than that of unsuccessfully infected gerbils. Eosinophil (Eos), basophil (Bas) and white blood cells (WBC) also have significant elevations (Table 2).

## 4. Discussion

*Strongyloides stercoralis* is a zoonotic parasite that is maintained in the laboratory primarily through its natural host, the beagle [3,12]. However, the beagle is costly for in vivo animal experiments. In a previous study, many small animals, such as rabbits and mice that are often used as laboratory animal models, had been attempted for infection with *S. stercoralis* [16]. However, so far, *S. stercoralis* infection has only been established in *M. unguiculatus* [17,18,19]. *S. stercoralis* from dogs as well as humans can produce stable infections in *M. unguiculatus* [22], suggesting that the gerbil is a good choice for modelling parasitic infections from either humans or dogs. In the present study, *S. stercoralis* completed its life cycle in *M. meridianus*, which has the closest phylogenetic relationship with *M. unguiculatus* being in the same genus [23]. Furthermore, regarding their living habits, *M. meridianus* need several times more water consumption than *M. unguiculatus* [27], which will increase the humidity of faeces on the ground and, in turn, the survival rate of larvae in faeces. From the perspective of species conservation, *M. meridianus* is considered a least concern species, while *M. unguiculatus* is a vulnerable species, as defined by the International Union for Conservation of Nature (IUCN). From the above analysis, *M. meridianus* may be more advantageous than *M. unguiculatus* for *S. stercoralis* research as a laboratory animal model of infections.

RNA interference cannot be performed in *S. stercoralis* [12]. In recent years, researchers have conducted functional studies of *S. stercoralis* genes by overexpressing loss-of-function proteins, but they are limited to only phenotypic observations of worms at developmental stages in vitro [14,15]. In addition, with the development and application of gene editing technology, CRISPR/Cas9 was successfully established in *S. stercoralis* in 2017 [34], and the defect of RNA interference in *S. stercoralis* has been effectively compensated, which is of great scientific value. However, it is a pity that the study of gene function is still limited to developmental stages of *S. stercoralis* in vitro due to the lack of suitable small animal models. The successful infection of the classic animal model of the *S. stercoralis*, the beagle, requires more than 3,000 iL3s to be used at the same time [12], but such a large number of transgenic worms cannot be obtained. In this study, *M. meridianus* only needed one-third of the worms to complete the infection and a sufficient number of *S. stercoralis* at different developmental stages was obtained for morphological observations. Although the acquisition of 1000 transgenic worms is still difficult, in this experiment, the infection efficiency in gerbils was 100%. After the optimization of the infection scheme, it is very likely a small amount of iL3 can successfully infect *M. meridianus* because gerbils are the natural host of *S. stercoralis,* and their body size is nearly a hundred times smaller than that of beagles. This study lays the foundation for the in-depth study of the developmental biology of *S. stercoralis*.

The functional study of genes is not only limited to the genes themselves, but it also requires a comprehensive analysis of the gene regulatory network, which requires a combination of multiple sequencing methods for research [35,36]. Sequencing experiments have high requirements for the quality and quantity of samples. A large number of PFs and larvae, including aL3s, were found in *M. meridianus* infected with *S. stercoralis* for 21 days. Necropsies of *M. meridianus* infected with 1000 iL3s had been done on day 21 post-infection because it was reported that L3s and PFs were both recovered at their peak on day 21 after their infection of steroid-treated *M. unguiculatus* [18]. More importantly, the number of PFs in the intestine of *M. meridianus* (126) is higher than that from *M. unguiculatus* (83.6 ± 27.6) [18]. Although the number of worms from different hosts varies, the morphologies of the worms are similar. The morphologies of PFs and aL3s from *M. meridianus* are very consistent with those from beagles and *M. unguiculatus* [14,18,37], which indicates that *S. stercoralis* could complete the parasitic life cycle in *M. meridianus*. At the same time, a large number of rhabditiform larvae (L1 and L2) were also collected from *M. meridianus*, and the PFs and rhabditiform larvae obtained from one animal model should facilitate developmental biology research of *S. stercoralis* using an omics approach.

Previous studies have shown that there were more PFs collected from male *M. unguiculatus* [18,22], while in the present study, more larvae were found in the faeces of females than in males for *M. meridianus*. For male and female *M. meridianus*, the ratio of the number of worms in tissues to that in the faeces is different [18,22]. Male *M. meridianus* may have a weaker ability to expel worms, resulting in an increased number of worms in the body and the production of a large number of eggs for reproduction, while female *M. meridianus* are better suited for excreting worms into the wild and spreading pathogens to other animals. In addition, male gerbils show a higher infection efficiency in *S. stercoralis* infections with *M. unguiculatus* under the same infection conditions [22], whereas there was no difference in the infection efficiency between female and male *M. meridianus*. This is possibly due to the fact that the differentiation requires infecting more *M. meridianus* than the six used in this study. *Meriones meridianus* is widely distributed in Northwest China [23]. The use of *M*. *meridianus* as an experimental animal is not as common as mice, so the commercialization of *M. meridianus* is very limited. For areas outside of Northwest China, the purchase of *M*. *meridianus* is difficult. There is a high mortality rate and high transportation costs for nearly 3 days of land transportation from Xinjiang to Wuhan. In addition, efforts to establish *M*. *meridianus* breeding in our own laboratory have not been successful. It is a pity that a sufficient number of *M. meridianus* were not used in this study to accurately determine the details of *M. meridianus* as an animal model of *S. stercoralis* infections. However, this study will promote the use of *M. meridianus* as animal models.

The blood physiology and serum biochemical indexes of *M. meridianus* changed significantly with the *S. stercoralis* infection. PFs, which primarily live in the intestinal mesentery, cause significant haemorrhages in the intestine and mild anaemia to the host [3,19]. This is consistent with the decreased red blood cells (RBC) and the constant Haematocrit (HCT) [38]. Alanine aminotransferase (ALT) is mainly distributed in liver cells. If the liver is damaged, the ALT in liver cells enters the bloodstream, and the level of ALT in the blood becomes elevated, which is the most common laboratory test for patients with chronic hepatitis. It is noted that when 1% of hepatocytes undergo necrosis, ALT in the serum increases 1-fold [39]. Compared with PFs, the damage of aL3s to the host is mainly caused by their migration into various organs [19]. Compared with the unsuccessfully infected *M. meridianus*, the ALT of successfully infected *M. meridianus* increased by 4.3 times, that is, a large number of liver cells died. This is also consistent with the lesions caused by *S. stercoralis* to *M. unguiculatus* [19], although there were no noticeable liver tissue lesions detected in *M. meridianus*. Low risks, such as no noticeable liver tissue lesions, to the host may allow the host to carry worms for long periods and reduce the number of experimental animals used. Small animal models capable of modelling some features of pathology should advance the understanding of the immunological mechanisms involved in host defence.

## 5. Conclusions

In the present study, the ability of *S. stercoralis* to establish an infection in immunosuppressed *M. meridianus* was assessed and confirmed, and the ability is different between female and male gerbils. *Strongyloides stercoralis* can infect *M. meridianus,* and the detection of *S. stercoralis* at different stages of development suggests that *S. stercoralis* could complete the entire life cycle in *M. meridianus.* In addition, the morphology of the worms collected from *M*. *meridianus* is the same as that of worms obtained from the beagle and *M. unguiculatus*. The blood parameters from *M. meridianus* after the infection is consistent with the symptoms of injury caused by *S. stercoralis*. In summary, this study successfully established *M. meridianus* as an animal model for studying *S. stercoralis* infections.

## Figures and Tables

**Table 1:** The body length and maximum diameter of *Strongyloides stercoralis* post-parasitic third-stage larvae (PP L3) and PFs from *Meriones meridianus*, *Meriones unguiculatus* and beagles.

Hosts	Stage	Body Length (mm) ^2^	Maximum Diameter (mm) ^2^
*M. meridianus*	PF	2.14 ± 0.263	0.039 ± 0.031
	PP L3	0.544 ± 0.181	0.019 ± 0.028 ^1^
*M. unguiculatus*	PF	2.12 ± 0.142	0.042 ± 0.016
	PP L3	0.521 ± 0.142	0.022 ± 0.032 ^1^
Beagle	PF	2.09 ± 0.212	0.042 ± 0.016
	PP L3	0.513 ± 0.162	0.018 ± 0.057 ^1^

^1^ The process of tableting and photographing causes the worms to be squeezed, which has a greater impact on the body width and maximum diameter of the worms. ^2^ Mean ± SD.

**Table 2:** Changes in physiological and biochemical parameters of *Meriones meridianus* infected with *Strongyloides stercoralis*.

Indicators	*Meriones meridianus*	
	Group 1 (Unsuccessfully Infected)	Group 4 (Successfully Infected)
ALT (U/L)	46	201
TG (mmol/L)	0.84	16.69
HCT (%)	28.3	31.6
RBC (10^12^/L)	6.13	4.68
Bas (%)	0.1	0.3
Eos (%)	0.4	0.8
WBC (10^9^/L)	3.8	4.5
PLT (10^9^/L)	268	1555

## Data Availability

Not applicable.
